# Ocular biometric parameters among 3-year-old Chinese children: testability, distribution and association with anthropometric parameters

**DOI:** 10.1038/srep29577

**Published:** 2016-07-07

**Authors:** Dan Huang, Xuejuan Chen, Qi Gong, Chaoqun Yuan, Hui Ding, Jing Bai, Hui Zhu, Zhujun Fu, Rongbin Yu, Hu Liu

**Affiliations:** 1 Department of Ophthalmology, The First Affiliated Hospital with Nanjing Medical University, Nanjing, 210029, China; 2 Maternal and Child Healthcare Hospital of Yuhua District, Nanjing, 210012 China; 3 Nanjing Medical University, Nanjing, 210029, China

## Abstract

This survey was conducted to determine the testability, distribution and associations of ocular biometric parameters in Chinese preschool children. Ocular biometric examinations, including the axial length (AL) and corneal radius of curvature (CR), were conducted on 1,688 3-year-old subjects by using an IOLMaster in August 2015. Anthropometric parameters, including height and weight, were measured according to a standardized protocol, and body mass index (BMI) was calculated. The testability was 93.7% for the AL and 78.6% for the CR overall, and both measures improved with age. Girls performed slightly better in AL measurements (*P* = 0.08), and the difference in CR was statistically significant (*P* < 0.05). The AL distribution was normal in girls (*P* = 0.12), whereas it was not in boys (*P* < 0.05). For CR1, all subgroups presented normal distributions (*P* = 0.16 for boys; *P* = 0.20 for girls), but the distribution varied when the subgroups were combined (*P* < 0.05). CR2 presented a normal distribution (*P* = 0.11), whereas the AL/CR ratio was abnormal (*P* < 0.001). Boys exhibited a significantly longer AL, a greater CR and a greater AL/CR ratio than girls (all *P* < 0.001).

A recent increase in myopia has been recorded in East Asia, with up to 90% of teenagers and young adults having myopia^1^. Approximately half of the young adults in the United States and Europe are also affected^1^. Studies in recent decades have recognized the importance of simple, reliable, and economical vision screening to detect vision-threatening conditions in children^2^,^3^,^4^. As recommended by the National Expert Panel, children aged 36 to 72 months should be screened annually (best practice) or at least once (accepted minimum standard)^5^.

The refractive state is dependent on the balance of changes in the axial length (AL) and the refractive components, including the cornea and the lens^6^, in which disturbances result in refractive errors. Non-contact partial coherence interferometry techniques, such as that used by IOLMaster, are more accurate and reproducible than A-scan ultrasonography in measuring ocular biometric parameters in school-age children^7^,^8^, and the reliability of IOLMaster has been validated in adults^9^,^10^. IOLMaster, a commonly used eye test has been considered to be applicable for preschool children aged 30 to 72 months. The Multi-Ethnic Pediatric Eye Disease Study (MEPEDS)^11^ and the Sydney Pediatric Eye Disease Study (SPEDS)^12^ have involved multi-ethnic populations, and the Strabismus, Amblyopia, and Refractive Error Study (STARS)^13^ has involved a Singapore Chinese population. However, children in Mainland China have not been deemed testable in a screening setting.

In past decades, the distributions of ocular biometric parameters in children have been infrequently reported in population-based studies using modern measurement techniques. A large proportion of these techniques have targeted school-children^14^,^15^,^16^,^17^,^18^,^19^, because of the poor patience and cooperation of preschool children. Monitoring the longitudinal changes of ocular biometric parameters in preschool children in large populations is important to identify the normal range of ocular biometric parameters and to understand the development of early-onset myopia.

In the past several decades, the height and weight of populations have increased^20^, as has the prevalence of myopia^21^, thus suggestting the possibility of a shared mechanism of action. Previous studies have demonstrated associations between body stature and AL, providing indirect or direct evidence for coordinated growth of the eye and body^22^,^23^,^24^,^25^,^26^. A study involving adult participants has revealed that a common set of genetic variants regulates body and eye growth^27^. However, the participants in previous studies have been older than 6 years^28^,^29^,^30^,^31^,^32^,^33^,^34^, and the relationship between anthropometry and ocular biometry remains unclear because of the absence of precise ocular biometric parameters for a large sample.

This cross-sectional and population-based study was performed to determine the testability of ocular biometric parameters by using IOLMaster in a screening setting, to describe the distribution of ocular biometric parameters, including the AL, the corneal radius of curvature (CR), and the AL/CR ratio, and to investigate the effect of anthropometric parameters, including height, weight and body mass index (BMI), on the ocular biometric parameters in Chinese preschool children aged 3 years old. This analysis is part of the ongoing prospective population-based Yuhua Pediatric Eye Disease Study (YPEDS).

## Results

### Study population

A total of 2,300 preschoolers were enrolled in this study, and 1,733 participated in ocular biometric examinations (75.3% responses rate). After checking the data, 45 children were excluded because of incomplete or missing personal information. Among the remaining 1,688 (73.4%) subjects, slightly more boys (907, 53.7%) than girls (781, 46.3%) were included. The mean age was similar between the boys (40.75 ± 3.33 months) and girls (40.73 ± 3.40 months) (*P* = 0.91).

### Testability of ocular biometric parameters by IOLMaster

**Figure 1:**
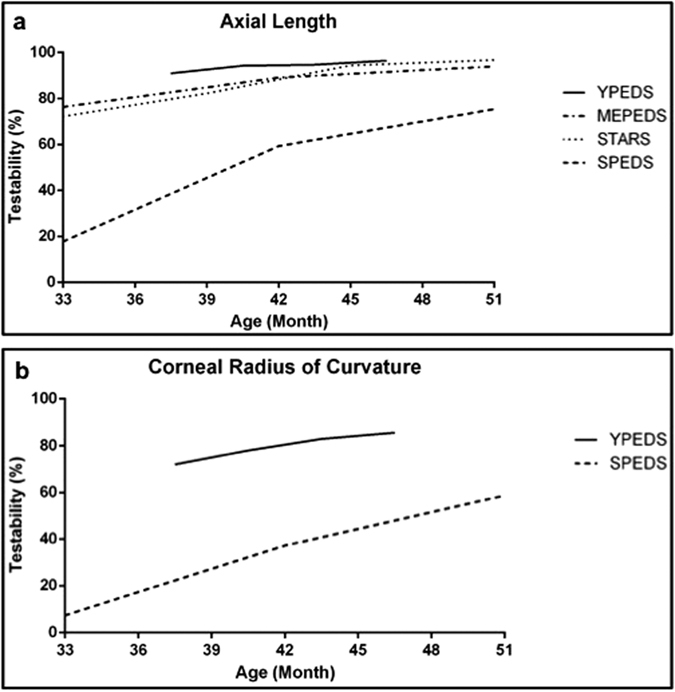
A comparison of the testability rates of AL and CR among the YPEDS, the MEPEDS, the STARS, and the SPEDS. (**a**) The total testability of AL using the IOLMaster was 93.7%, which is superior to the values reported by the MEPEDS (89.1%)^11^, the STARS (88.4%)^13^, and the SPEDS (59.3%)^12^. (**b**) The SPEDS reported a testability for CR (37.2%) in the 36- to 47-month-old group^12^, that is evidently lower than the 78.6% we obtained.

Table 1 details the testability of the ocular biometric parameters. In our sample, the AL was more testable (1,582, 93.7%) than the CR (1,326, 78.8%). Figure 1a,b compare the testability rates of the AL and CR in our study and in the MEPEDS^11^, the STARS^13^, and the SPEDS^12^. The testability of AL ranged from 91.2% in the 36- to 39-month-old group to 96.6% in the 45- to 48-month-old group. No gender-related differences in testability were observed in any age group. The testability of CR ranged from 72.6% in the 36- to 39-month-old group to 85.6% in the 45- to 48-month-old group. The testability for both measurements improved with age (all *P* for trends < 0.05). Girls performed slightly better in AL measurements (94.8 versus 92.8%, *P* = 0.08), and the difference in CR measurements between girls and boys was statistically significant (80.7 versus 76.7%, *P* < 0.05). The girls in the 39- to 42-month-old group were more testable than the boys for CR (83.1 versus 74.3%, *P* < 0.05). No gender-related differences in testability were found for the other age groups.

### Distribution

Statistically significant differences were found between the two eyes for the AL (21.88 mm in the right eyes; 21.86 mm in the left eyes; *P* < 0.001) and CR1 (7.897 mm in the right eyes; 7.903 mm in the left eyes; *P* < 0.05), whereas CR2 (7.657 mm in the right eyes; 7.657 mm in the left eyes; *P* = 0.948) and the AL/CR ratio (2.814 in the right eyes; 2.813 in the left eyes; *P* = 0.073) were similar.

Given the high Pearson correlations between the right and left eyes for ocular biometric parameters (AL, 0.969; CR1, 0.963; CR2, 0.960; AL/CR ratio, 0.942), only the results of the right eyes are presented. In total, 1,605 children provided ocular biometric parameters for the right eyes. Among these children, 1,603 presented AL values, including 854 boys (53.3%) and 749 girls (46.7%) with a similar mean age (40.82 ± 3.34 versus 40.77 ± 3.40 months, *P* = 0.75), and 1,479 children showed CR values, including 777 boys (52.6%) and 702 girls (47.4%) with a similar mean age (40.90 ± 3.34 versus 40.81 ± 3.39 months, *P* = 0.59). The AL/CR ratio was calculated for 1,477 children because two children were missing AL values.

**Figure 2:**
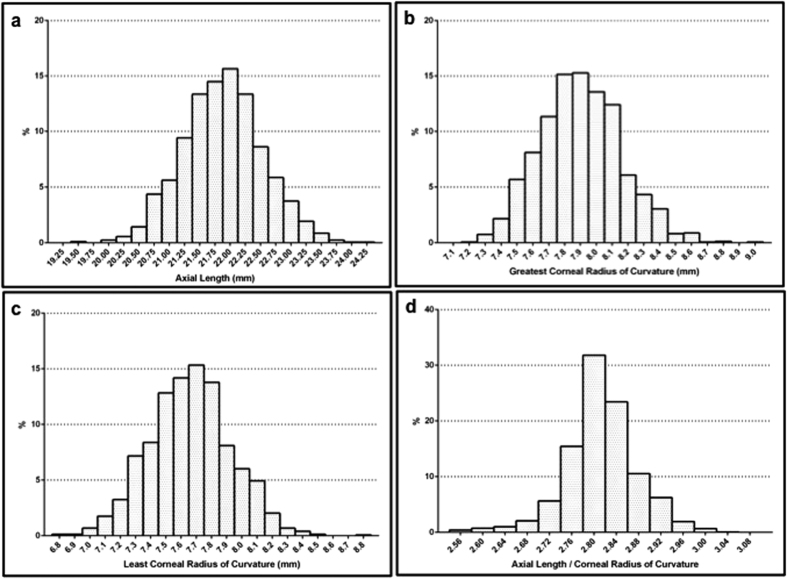
The distribution of the ocular biometric parameters in the right eyes, with the values on the x-axis for all of the figures representing the midpoint of the corresponding bin. (**a**) The distribution of AL was normal for the total sample and the girls but was abnormal for the boys. (**b**) The distribution of CR1 among children aged 3 years was 7.90 ± 0.26 mm and was normally distributed. (**c**) The distribution of CR2 was also normal for the total subjects (7.66 ± 0.27 mm). (**d**) The present results clearly showed that a ratio of less than 3.0 was associated with hypermetropia because nearly all children aged 3 years who were enrolled in the YPEDS belonged to this category, with a value of 2.81 ± 0.06.

Table 2 and Fig. 2 show the distribution of ocular biometric parameters in the right eyes, with the values on the x-axis for all of the figures representing the midpoint for the corresponding bin. The distribution of AL in the girls was normal (*P* = 0.12), whereas the distribution in the boys was not (*P* < 0.05). For CR1, all of the subgroups presented normal distributions (*P* = 0.16 for boys; *P* = 0.20 for girls), but the distribution varied when the subgroups were combined (*P* < 0.05). CR2 presented a normal distribution (*P* = 0.11), whereas the AL/CR ratio had an abnormal distribution (*P* < 0.001). Compared with the girls, the boys exhibited a significantly longer AL (22.14 versus 21.58 mm), a larger CR1 (7.98 versus 7.81 mm) and CR2 (7.74 versus 7.57 mm), and a slightly higher AL/CR ratio (2.82 versus 2.81) (all *P* < 0.001).

Table 3 shows the distribution of ocular biometry according to the categories of refractive status. Children in group A (SE ≤ −1.00 D) and group B (SE between −1.00 D and +1.00 D) had a similar AL (21.91 ± 0.71 mm in group A, 21.92 ± 0.63 mm in group B) and AL/CR ratio (2.82 ± 0.07 in group A, 2.82 ± 0.06 mm in group B), and group C (SE ≥ 1.00 D) had the shortest AL (21.70 ± 0.69 mm) and the smallest AL/CR ratio (2.79 ± 0.07). All of the children had a similar CR1 (7.91 ± 0.26 mm in group A, 7.90 ± 0.26 mm in group B, 7.91 ± 0.27 mm in group C). The CR2 was smaller in group A (7.62 ± 0.30 mm) than in groups B (7.66 ± 0.27 mm) and C (7.67 ± 0.27 mm).

### Association with birth parameters

The anthropometric characteristics of the children are shown in Table 4. The mean height, weight, and BMI were 100.18 cm, 15.81 kg, and 15.70 kg/m^2^, respectively. Compared with the girls, the boys were taller and heavier and had a higher BMI (100.91 versus 99.33 cm; 16.21 versus 15.34 kg; 15.87 versus 15.50 kg/m^2^; all *P* < 0.001).

The correlations of age, height, weight and BMI with the ocular biometric parameters in the right eyes, including AL, CR, and the AL/CR ratio, are shown in Table 5. AL, CR1, CR2, and the AL/CR ratio were positively correlated with both height (*r* = 0.297; *r* = 0.201; *r* = 0.204; *r* = 0.093; respectively, all *P* < 0.001), and weight (r = 0.246; r = 0.183; r = 0.150; r = 0.073; respectively, all P < 0.001). Age showed a weak positive correlation with AL and the AL/CR ratio (*r* = 0.095; *r* = 0.127, both *P* < 0.001). BMI exhibited a weak positive correlation with AL and CR1 (*r* = 0.104; *r* = 0.092, both *P* < 0.001).

The regression models are shown in Table 6. In the table, each value represents the result of a separate regression model. After controlling for age in months, gender, and weight, a child that is taller by 10 cm taller is expected to have a 0.30 mm increase in AL, a 0.10 mm increase in CR1, and a 0.11 mm increase in CR2 (all *P* < 0.001). Weight had no effect on AL, CR1, and CR2 after controlling for age in months, gender, and height. After controlling for age in months and gender, a child with a 10-unit increase in BMI is expected to have a 0.23 mm increase in AL and a 0.09 mm increase in CR1. No significant associations were found between the AL/CR ratio and any anthropometric parameters.

## Discussion

In this study, the total testability of the AL by using the IOLMaster was 93.7%, which is superior to the values reported by the MEPEDS (89.1%)^11^, the STARS (88.4%)^13^, and the SPEDS (59.3%)^12^ in the 36- to 47-month-old group (Fig. 1a). Only the SPEDS had previously reported testability for CR (37.2%) in 36- to 47-month-old children^12^, and the value is evidently lower than the 78.6% reported in our study (Fig. 1b). To gather accurate data for subsequent analysis, we provided a restricted definition of testability, that is, obtaining a weighted mean value provided by the IOLMaster system after a minimum of five attempts for AL and obtaining both readings (CR1 and CR2) for CR after a minimum of three attempts. A minimum of two AL readings (mainly < 0.1 mm difference) in both eyes was considered successful testing in the three other studies. In our experience, almost all children would be identified as testable according to the more lenient definition of the three other studies. We performed an IOLMaster examination for approximately 1,700 preschool children within a month in a screening setting, whereas the duration of the three other studies exceeded one year. However, our examination was performed under non-cycloplegic conditions, which made the children more cooperative.

Age was a critical affecting testability in the 30- to 72-month-old group in all prior studies, and testability was found to be strongly age dependent in a narrower age range in our study. The SPEDS reported an association between the sequence of testability (AL > CR > anterior chamber depth) and testing (AL first, followed by CR, and then anterior chamber depth). We also observed a higher testability of AL than CR, suggesting the influence of the duration and order of testing because of the poor patience of preschool children. Moreover, prior studies have evaluated the influence of sex on testability. Sex was not a significant predictor of testability for AL in the STARS and the SPEDS; however, the MEPEDS found that girls exhibited a higher testability than boys in the 36- to 47-month-old group (94 versus 85%, *P* < 0.0001). Girls performed slightly better than boys in CR measurements in the SPEDS (*P* = 0.051)^12^. In the present study, girls performed better in CR measurements (80.7 versus 76.7%, *P* < 0.05), particularly in the 39- to 42-month-old group (83.1 versus 74.3%, *P* < 0.05). This result may be attributed to the higher patience of girls than boys in overcoming the obstacle of duration and the order of testing.

AL measured by interferometry was slightly longer than that obtained by ultrasound possibly because the IOLMaster measurement is noncontact and is assessed along the visual axis with the aid of a fixation beam^35^.

Decreasing hyperopia during the transition from infancy to adulthood has been confirmed. Rapid eye growth occurs during the infantile phase. In this phase, the eye must compensate by approximately 20 D for an increase of 5 mm in the AL, with adult dimensions nearly reached within 2 years. A slower phase follows when the eye must to compensate for approximately 3 D, which is attributable to a 1 mm increase in AL^36^. A sample of 255 preschool children aged 2 to 6 years in Hong Kong has been found to exhibit an AL of 21.99 ± 0.77 mm^37^. In Singaporean children aged 4 to 6 years, boys have been found to show a longer AL (22.62 ± 0.75 mm) than girls (22.08 ± 0.71 mm) in a sample of 469 subjects^38^. We provided a relatively precise value of the mean AL for Chinese children aged 3 years (21.88 ± 0.65 mm; 22.14 ± 0.59 mm for boys; 21.58 ± 0.58 mm for girls). These values approximate adult dimensions, and the distribution of AL was normal in the total sample and in girls but was abnormal in boys (Fig. 2a). Prior studies have reported mean AL values in school children aged 6 to 7 years ranging from 22.32 to 23.40 mm, with an essentially normal distribution^14^,^16^,^17^,^18^,^19^,^39^.

Previous studies have suggested that the CR becomes flatter to compensate for increasing AL, and the failure of this process results in myopia^40^. The CR varies slightly in children aged 5 to 14 years^19^. In the present study, children aged 3 years presented a CR1 of 7.90 ± 0.26 mm and a CR2 of 7.66 ± 0.27 mm, with a normal distribution (Fig. 2b,c). Thses values are similar to those in previous studies, ranging from 7.73 to 7.88 mm in adolescents^14^,^39^,^41^, and are consistent with the consensus that a major proportion of eye growth is achieved within 2 years.

Given the compensatory adjustment of optical components involving interactions between AL and CR, the AL/CR ratio has been found to explain the total variance in diopter better than AL alone, with a criterion of 3.0 for the AL/CR ratio (based on the horizontal radius) separating eyes that have become myopic from those that remain emmetropic^41^,^42^. This finding has been demonstrated by studies in children with predominantly hyperopic refraction^18^,^39^, and myopic refraction^43^,^44^. It has been reported that the variance in the AL/CR ratio could account for the 80% of the school children and the 84% of the young adults^39^,^45^. A 14-year longitudinal study of 469 schoolchildren has demonstrated increases in the AL/CR ratio as myopia progresses and then stabilizes^44^. A study of 3,922 children has found an optimal cutoff value of >2.99^41^, which is near the cutoff value (3.0) found by Grosvenor and Goss^42^, The AL/CR ratio may be a better method for screening for children with myopia than the traditional uncorrected visual acuity method used in most screening, and the combination of the two methods may increase sensitivity without significantly decreasing specificity^41^.

Recently, Valencia F and coworkers described the distribution of the AL/CR ratios of 349 3-year-old children, which were all less than 3 (2.89 for myopes, 2.85 for emmetropes, and 2.79 for hyperopes), results concluded that the AL/CR ratio is correlated with cycloplegic refraction in myopes, but not in emmetropes and hyperopes^46^. Compared with cycloplegic refraction, the usefulness of AL/CR is limited but might be the next best reflection of their refractive error when one is unable to obtain cycloplegic refraction in the setting of screening^46^. Children aged 3 years who were enrolled in the YPEDS had a mean AL/CR ratio of 2.81 ± 0.06 (Fig. 2d). As classified into three groups according to refractive status, the AL/CR ratios were 2.82 ± 0.07 for group A, 2.82 ± 0.06 for group B and 2.79 ± 0.07 for group C. The distribution of refraction reportedly becomes more peaked with age, with an initial extremely broad distribution in neonates^47^. A leptokurtic distribution of the AL/CR ratio was observed in the present study, demonstrating that essential remodeling of the ocular components had already occurred in preschool children aged 3 years.

Eye growth when body stature is increasing suggests the potential for a shared mechanism of action. Limited studies have confirmed the association of ocular biometry and refraction with weight and BMI^28^. In the present study, the positive correlation between weight and ocular biometry disappeared after controlling for age, sex and height. In contrast, height has been thought to present the closest correlation to ocular biometry. A study of 565 pairs of twins has revealed that 89% of the phenotypic correlation between AL and height is a result of shared genetic factors^27^.

In school-age children, a strong association has been reported between height and AL and CR. Tall children have eyes with a longer AL, deeper vitreous chambers, and flatter corneas than short children^28^,^29^. Different relationships have been observed between AL and CR with regard to the timing of their respective associations with body growth trajectory, suggesting that early growth is more important in determining CR in young adulthood than for determining AL^33^. After controlling for age in months, sex and weight, a 10 cm taller child is expected to exhibit a 0.30 mm increase in AL, a 0.10 mm increase in CR1, and a 0.11 mm increase in CR2, indicating a positive correlation between height and AL and CR during preschool age.

Tall Singapore Chinese children are susceptible to myopia^28^, However, several studies have not found an association between height and refraction^29^,^33^,^34^, despite the correlation between AL and height. This finding indicates the influence of other ocular components, such as the cornea or lens. Similarly, the present results showed that no anthropometric parameters were correlated with the AL/CR ratio at preschool age. After comparing the growth trajectories (between birth and 10 years), refractive errors (aged 11 to 15 years), and AL (aged 15 years), a study in the UK has concluded that shared growth mechanisms up to the age of 10 years contribute to the scaling of the eye and body size and minimally to the development of myopia^33^. A recent animal model of myopia has suggested that genetic variants influencing both body and eye size may differ from those that confer susceptibility to myopia^48^,^49^. Another study has suggested that the genetic variants that influence height in adulthood might not be predictive of myopia development to any useful extent at an individual level^33^.

Despite the strengths of a large sample size and a specific age norm unique, this study presents certain limitations. Firstly, the greastest limitation of this study is that we used noncycloplegic refraction because of the parental refusing and the poorer patience and cooperation after cycloplegia especial in younger children. Second, most ocular biometrics including AL, CR, anterior chamber distance (ACD), lens thickness and vitreous chamber depth contribute importantly to the emmetropization process, and the measurements for LT and VCD could not be obtained directly by using an IOLmaster, unless applying ultrasonography such as an A-scan ultrasound. However, the A-Scan ultrasound is not suitable for preschool children screening for it is a type of contact partial coherence interferometry technique. We did not use the ACD because of the strong stimulation of harsh white light emitted by the IOLMaster machine with only 1/3 of children able to complete the testing for ACD in the pre-screening test, thus reducing the breadth of our study. Furthermore, the response ratio rate was only 73.4%, which weakens the representativeness of our data. Finally, a certain bias may exist toward families with parents who care more about eye growth in children.

In conclusion, ocular biometric parameters were testable in a screening setting for preschool children aged 3 years despite the limitation of age. Futhermore, the study provided definitive ocular biometric parameter data in a representative population of preschool children aged 3 years in Eastern China. The AL and CR were normally distributed with statistically significant gender differences, and a peaked distribution of the AL/CR ratio was present in this predominantly hyperopic 3-year-old population.

## Methods

### Study design and population

The YPEDS is a population-based vision screening study that was conducted from July 2015 to August 2015; the YPEDS sought to establish a systematic database on refraction, visual acuity, ocular biometric parameters, ocular position and other ophthalmic measures, to explore the development rule of vision and to estimate the burden of common pediatric ocular disorders of preschool children aged 3–6 years in the Yuhua District, Nanjing, China. The Yuhua District is one of the 11 municipal districts of Nanjing and has a medium socioeconomic status in Eastern China; it has a permanent population of 413,000 (2012), which ensures the representativeness of this area. The YPEDS used similar inclusion and exclusion criteria as the MEPEDS^50^: (1) age five months to 70 months on the day of the household screening, and (2) parent or legal guardian confirmation that the participant resides in one of the selected MEPEDS census tracts. With confirmation from the parents or legal guardians that the participant resides in Yuhua District, all of the children who were born between September 2011 and August 2012 and were about to enter a kindergarten in Yuhua District were invited to participate in this study and to undergo a further vision examination in addition to a comprehensive compulsory health examination.

This study was approved by the Ethics Committee of Nanjing Medical University and followed the tenets of the Declaration of Helsinki. Written informed consent was obtained from the parents or legal representatives of all the participating children.

### Examination

Comprehensive eye examinations were performed by a team of two optometrists and two ophthalmologists who were trained and certified in using standardized study protocols, as described in the MEPEDS previously^50^. Basic participant information including name, gender, nationality, date of birth, and exam date, was recorded during the clinical visit. The examinations included anthropometric parameters, distance visual acuity (using an HOTV VA chart at a distance of 3 m), anterior segment examination, autorefraction (R-F10, Cannon, Tokyo, Japan), photorefraction, cover test at distant and near fixation, ocular motility, fundus examination and ocular biometric parameters.

Height in centimeters was measured without shoes, and weight in kilograms was measured using a standard scale weighing machine that was calibrated prior to the eye examinations. Ocular biometric parameters were measured by the same operator using an IOLMaster (Carl Zeiss Meditec, Jena, Germany; V5.5.0.0062) under non-cycloplegic conditions in August 2015. The children were seated comfortably under normal room lighting. Children who wore glasses were instructed to remove their spectacles before measurement. After positioning their heads on the chin rest, the participants were instructed to look at an internal fixation target during the examination. The right eye was tested first.

AL measurements were attempted a minimum of five times until the IOLMaster system provided a weighted mean value on all children for each eye. The reliability of each AL measurement was assessed using the signal-to-noise ratio (SNR), and the reading was accepted if the SNR ≥ 2.0. CR measurements were conducted for at least three times in the principal meridians to yield the greatest corneal radius of curvature (CR1), and the lowest corneal radius of curvature (CR2). The mean CR was calculated as the average of CR1 and CR2. The AL/CR ratio was defined as the AL divided by the mean CR.

Testability was defined as the ability to successfully complete IOLMaster tests in both eyes. For AL, testability was defined as obtaining a weighted mean value from the IOLMaster instrument. For CR, testability was defined as obtaining both readings (CR1 and CR2). Missing or incomplete results were identified as not testable. The spherical equivalent (SE) refractive error was calculated as the sphere power plus one half of the absolute cylinder power. The children were classified into three groups according to the refractive status of the right eyes (if the ocular biometrics of the two eyes were highly correlated) without cycloplegia. Group A was defined as SE myopia of ≤−1.00 diopter (D). Group B was defined as SE emmetropia between −1.00 D and +1.00 D. Group C was defined as SE hyperopia ≥+1.00 D.

### Data Analysis

Statistical analyses were performed using the Statistical Product and Service Solutions (SPSS) for Windows V.7.0 software (V.22.0, IBM, China). The measures are presented as the mean ± SD. All of the probabilities quoted are two-sided and were considered to be statistically significant at less than 0.05. All of the confidence intervals (CIs) are 95%.

For the analysis of testability, the linear-by-linear association test was used to calculate the significance of the trend values for each testing modality with increasing age groups. The CIs for the proportion of testable children were calculated from the normal approximation of the binomial distribution. The comparisons of the testable proportion in the age and sex groups were calculated using χ^2^ tests. We used multiple logistic regression models to compare testability measures between sex groups (adjusted for age).

To describe the distribution of ocular biometry, the correlation between the right and left eye was analyzed by Pearson’s correlation, and only the results of the right eyes were presented when the two eyes were highly correlated. Measures of spread, including kurtosis and skewness, were derived. The distributions of the ocular biometric parameters were tested for normality using the Kolmogorov-Smirnov test. Sex-specific differences in the ocular biometric parameters were compared by multivariate models.

Only the results of the right eyes were used in the analysis of the association with the anthropometric parameters. The correlation of the ocular biometric parameters with the anthropometric parameters and age was analyzed using Pearson’s correlation. Multiple linear regression models were constructed. The ocular biometric components, including AL, CR, and the AL/CR ratio, were the dependent variables, whereas age, gender, and the anthropometric parameters, including height, weight, and BMI, were the independent variables. The models were adjusted as follows: if height was the independent variable, the factors were adjusted for age, gender, and weight; if weight was the independent variable, the factors were adjusted for age, gender, and height; if BMI was the independent variable, the factors were adjusted for age and gender.

## Additional Information

**How to cite this article**: Huang, D. *et al*. Ocular biometric parameters among 3-year-old Chinese children: testability, distribution and association with anthropometric parameters. *Sci. Rep.* **6**, 29577; doi: 10.1038/srep29577 (2016).

## Figures and Tables

**Table 1:** Testability by Age and Sex for the IOLMaster.

**Classification**	**Axial length**		**Corneal radius of curvature**	
	**Number tested/total**	**%**	**Number tested/total**	**%**
Age in months				
36 to <39	493/542	91.0 (88.5–93.4)	390/542	72.0 (68.2–75.7)
39 to <42	428/454	94.3 (92.1–96.4)	354/454	78.0 (74.1–81.8)
42 to <45	360/380	94.7 (92.5–97.0)	315/380	82.9 (79.1–86.7)
45 to <48	301/312	96.5 (94.4–98.5)	267/312	85.6 (81.7–81.7)
*P* for trend^a^		<0.05		<0.0001
Sex				
Boys	842/907	92.8 (91.2–94.5)	696/907	76.7 (74.0–79.5)
Girls	740/781	94.8 (93.2–96.3)	630/781	80.7 (77.9–83.4)
*P* value^b^		0.08		<0.05
Total	1582/1688	93.7 (92.6–94.9)	1326/1688	78.6 (76.6–80.5)

Data in parentheses represent the 95% confidence interval.

^a^For increasing age.

^b^Adjusted for age.

**Table 2:** General Distribution of Ocular Biometric Parameters.

**Classification**	**Mean**	**SD**	**Median**	**Range**	**Kurtosis**	**Skewness**	**K-S**	***P*** value*
AL (mm)								<0.001
Total (1603)	21.88	0.65	21.88	19.48 to 24.17	0.066	0.020	0.12	
Boy (854)	22.14	0.59	22.13	20.38 to 24.17	0.152	0.074	<0.05	
Girl (749)	21.58	0.58	21.58	19.48 to 23.23	0.123	0.002	0.20	
CR1 (mm)								<0.001
Total (1479)	7.90	0.26	7.88	7.18 to 8.98	0.068	0.235	<0.05	
Boy (777)	7.98	0.25	7.97	7.34 to 8.98	0.154	0.235	0.16	
Girl (702)	7.81	0.23	7.81	7.18 to 8.63	−0.017	0.266	0.20	
CR2 (mm)								<0.001
Total (1479)	7.66	0.27	7.66	6.83 to 8.79	0.043	0.080	0.11	
Boy (777)	7.74	0.26	7.74	6.87 to 8.79	0.120	0.040	0.20	
Girl (702)	7.57	0.25	7.57	6.83 to 8.41	0.085	0.083	0.20	
AL/CR ratio								<0.001
Total (1477)	2.81	0.06	2.81	2.56 to 3.05	0.855	−0.217	<0.001	
Boy (776)	2.82	0.06	2.82	2.56 to 3.01	0.862	0.083	<0.05	
Girl (701)	2.81	0.06	2.81	2.58 to 3.05	0.882	−0.266	<0.05	

AL, axial length; CR1, greatest corneal radius of curvature; CR2, least corneal radius of curvature; AL/CR ratio, axial length/mean corneal radius of curvature ratio.

K-S, Kolmogorov-Smirnov test for normality.

^*^Comparison between sex groups.

**Table 3:** Distribution of Ocular Biometric Parameters by Refraction Status without Cycloplegia.

**Ocular biometry**	**Group A**	**Group B**	**Group C**
	**n = 134**	**n = 1177**	**n = 294**
AL (mm)	21.91 ± 0.71	21.92 ± 0.63	21.70 ± 0.69
CR1 (mm)	7.91 ± 0.26	7.90 ± 0.26	7.91 ± 0.27
CR2 (mm)	7.62 ± 0.30	7.66 ± 0.27	7.67 ± 0.27
AL/CR ratio	2.82 ± 0.07	2.82 ± 0.06	2.79 ± 0.07

Group A, SE ≤ −1.00 D; Group B, SE between −1.00 D and +1.00 D; Group C, SE ≥+1.00 D; AL, axial length; CR1, greatest corneal radius of curvature; CR2, least corneal radius of curvature; AL/CR ratio, axial length/mean corneal radius of curvature ratio.

Data are presented as mean ± SD.

**Table 4:** Characteristics of Anthropometric Parameters.

**Classification**	**Mean**	**SD**	**Median**	**Range**	***P*** value*
Height (cm)					<0.001
Total (1605)	100.18	4.31	100.0	86.4 to 115.8	
Boy (855)	100.91	4.30	101.0	86.9 to 115.8	
Girl (750)	99.33	4.17	99.3	86.4 to 114.5	
Weight (kg)					<0.001
Total (1605)	15.81	2.34	15.5	10.6 to 31.8	
Boy (855)	16.21	2.38	15.9	11.5 to 31.8	
Girl (750)	15.34	2.21	15.0	10.6 to 30.0	
BMI (kg/m^2^)					<0.001
Total (1605)	15.70	1.66	15.46	11.16 to 31.80	
Boy (855)	15.87	1.69	15.59	11.31 to 31.80	
Girl (750)	15.50	1.60	15.23	11.16 to 30.92	

BMI, body mass index.

^*^Comparison between sex groups.

**Table 5:** Correlation of Age and Anthropometric Parameters with the Ocular Biometric Parameters.

	**Pearson Correlation Coefficient**			
	**Age (Month)**	**Height (cm)**	**Weight (kg)**	**BMI (kg/m^2^)**
AL (mm)	0.095^**^	0.297^**^	0.246^**^	0.104^**^
CR1 (mm)	−0.020	0.201^**^	0.183^**^	0.092^**^
CR2 (mm)	0.023	0.204^**^	0.150^**^	0.0 46
AL/CR ratio	0.127^**^	0.093^**^	0.073^**^	0.026

AL, axial length; CR1, greatest corneal radius of curvature; CR2, least corneal radius of curvature; AL/CR ratio, axial length/mean corneal radius of curvature ratio; BMI, body mass index.

^*^P < 0.05, **P < 0.001.

**Table 6:** Linear Regression Models of Ocular Biometry by Anthropometric Parameters.

	**AL (mm)**		**CR1 (mm)**		**CR2 (mm)**		**AL/CR ratio**	
	**Regression Coefficient**	**R**^**2**^	**Regression Coefficient**	**R**^**2**^	**Regression Coefficient**	**R**^**2**^	**Regression Coefficient**	**R**^**2**^
Height (cm)								
Total	0.030 (0.020 to 0.040)^**^	0.235	0.010 (0.005 to 0.014)^**^	0.133	0.011 (0.007 to 0.016)^**^	0.126	0.00004 (−0.001 to 0.001)	0.029
Boys	0.032 (0.018 to 0.045)^**^	0.063	0.011 (0.005 to 0.017)^**^	0.033	0.013 (0.007 to 0.019)^**^	0.028	−0.0002 (−0.001 to 0.001)	0.019
Girls	0.028 (0.013 to 0.043)^**^	0.053	0.008 (0.001 to 0.014)^*^	0.037	0.009 (0.003 to 0.016)^*^	0.029	0.001 (−0.001 to 0.002	0.016
Weight (kg)								
Total	0.009 (−0.008 to 0.025)	0.235	0.004 (−0.003 to 0.012)	0.133	−0.002 (−0.010 to 0.005)	0.126	0.001 (−0.001 to 0.002)	0.029
Boys	0.006 (−0.016 to 0.028)	0.063	0.001 (−0.009 to 0.011)	0.033	−0.007 (−0.017 to 0.003)	0.028	0.002 (−0.001 to 0.004)	0.019
Girls	0.012 (−0.014 to 0.038)	0.053	0.009 (−0.002 to 0.020)	0.037	0.004 (−0.007 to 0.016)	0.029	−0.001 (−0.004 to 0.002)	0.016
BMI (kg/m^2^)								
Total	0.023 (0.006 to 0.040)^*^	0.199	0.009 (0.0002 to 0.017)^*^	0.106	0.002 (−0.005 to 0.010)	0.102	0.001 (−0.001 to 0.003)	0.028
Boys	0.019 (−0.005 to 0.042)	0.014	0.005 (−0.005 to 0.016)	0.002	−0.004 (−0.014 to 0.007)	0.001	0.002 (−0.001 to 0.004)	0.019
Girls	0.029 (0.002 to 0.055)^*^	0.015	0.014 (0.003 to 0.025)^*^	0.011	0.010 (−0.001 to 0.021)	0.005	−0.001 (−0.003 to 0.002)	0.015

AL, axial length; CR1, greatest corneal radius of curvature; CR2, least corneal radius of curvature; AL/CR ratio, axial length/mean corneal radius of curvature ratio; BMI, body mass index. Data in parentheses represent the 95% confidence interval. *P < 0.05, **P < 0.001.
